# Categorizing diffuse parenchymal lung disease in children

**DOI:** 10.1186/s13023-015-0339-1

**Published:** 2015-09-25

**Authors:** Matthias Griese, Armin Irnstetter, Meike Hengst, Helen Burmester, Felicitas Nagel, Jan Ripper, Maria Feilcke, Ingo Pawlita, Florian Gothe, Matthias Kappler, Andrea Schams, Traudl Wesselak, Daniela Rauch, Thomas Wittmann, Peter Lohse, Frank Brasch, Carolin Kröner

**Affiliations:** Department of Pediatric Pneumology, Dr. von Haunersches Kinderspital, University of Munich, German Center for Lung Research, Lindwurmstraße 4, 80337, Munich, Germany; Praxis für Humangenetik, CeGaT GmbH, Tübingen, Germany; Department of Pathology, Academic Teaching Hospital Bielefeld, Bielefeld, Germany

**Keywords:** Childhood interstitial lung disease, chILD, Diffuse parenchymal lung disease, Rare pediatric lung disease, Categorization, Register, Registry

## Abstract

**Background:**

Aim of this study was to verify a systematic and practical categorization system that allows dynamic classification of pediatric DPLD irrespective of completeness of patient data.

**Methods:**

The study was based on 2322 children submitted to the kids-lung-register between 1997 and 2012. Of these children 791 were assigned to 12 DPLD categories, more than 2/3 belonged to categories manifesting primarily in infancy. The work-flow of the pediatric DPLD categorization system included (i) the generation of a final working diagnosis, decision on the presence or absence of (ii) DPLD and (iii) a systemic or lung only condition, and (iv) the allocation to a category and subcategory. The validity and inter-observer dependency of this workflow was re-tested using a systematic sample of 100 cases.

**Results:**

Two blinded raters allocated more than 80 % of the re-categorized cases identically. Non-identical allocation was due to lack of appreciation of all available details, insufficient knowledge of the classification rules by the raters, incomplete patient data, and shortcomings of the classification system itself.

**Conclusions:**

This study provides a suitable workflow and hand-on rules for the categorization of pediatric DPLD. Potential pitfalls were identified and a foundation was laid for the development of consensus-based, international categorization guidelines.

**Electronic supplementary material:**

The online version of this article (doi:10.1186/s13023-015-0339-1) contains supplementary material, which is available to authorized users.

## Background

Childhood interstitial lung diseases (ILD) represent a large spectrum of individually rare diffuse parenchymal lung diseases (DPLD), prevalent in children of all ages [1–3]. They comprise more than 200 different disease entities which are treated by pediatricians and general practitioners in general and specialized (children´s) hospitals. Due to the similarity of symptoms it is often difficult to differentiate these rare patients from children with more common respiratory diseases [4]. Clinical presentation of the disease may further be blurred by recurrent infections or allergies. Childhood DPLD may thus easily be underdiagnosed. Correct classification of all patients is however indispensable for the appropriate treatment, for a better understanding of the underlying pathophysiology, for the identification of biomarkers and for long-term studies and cohort investigations.

Several categorization systems of childhood DPLD have been proposed over time [1, 5–7]. The majority of the recent systems are based on lung histology, related to the study by Deutsch et al. [1], which classifies the broad spectrum of patients into eight disease categories containing various diagnoses [1]. The categorization system has in the meantime been expanded to the entire pediatric age range [6] and has been shown useful for pathological studies [7]. In a single center study the system was also used for cases not diagnosed by biopsy [8].

Aim of this study was to verify a systematic and practical categorization system that allows dynamic classification of pediatric DPLD irrespective of completeness of patient data. The work-flow and validity of the categorization system was tested, basis were all cases submitted to the kids-lung-register (KLR) between 1997 and 2012 [2]. The kids-lung-register is, an open, non-profit register for rare lung diseases in childhood and adolescence (www.kids-lung-register.eu). On average 147 children with lung diseases per year are referred to the kids-lung-register for consultation and laboratory services from diverse European centers. Based on the kids-lung-register a European management platform was established in 2013 for childhood interstitial lung diseases (http://www.klinikum.uni-muenchen.de/Child-EU/en/index.html) comprising 10 academic partners from 5 European countries.

## Methods

DPLD are entities originating from abnormalities of lung interstitial tissue components. These structures in the periphery of the lungs include the alveolar epithelium, the vessel endothelium and the tissues between these structures. More centrally they include peribronchiolar and peribronchial tissues [9]. Airways may be involved secondary in the disease process. DPLD disorders more prevalent in infancy (A) and disorders occurring at all ages (B) are differentiated. Diseases which affect the parenchymal tissue, but are localized gross structural abnormalities of the lungs, either congenital (C1) or acquired (C2) are not classified as DPLD. Further separated are disorders which primarily affect the airways (airway disorders (D)), the pleural tissues (pleural diseases (E)), diseases caused by lung infections (F) or neoplasms (G), which may also involve the parenchyma.

### Workflow for patient categorization during routine operation of the KLR

**Fig. 1:**
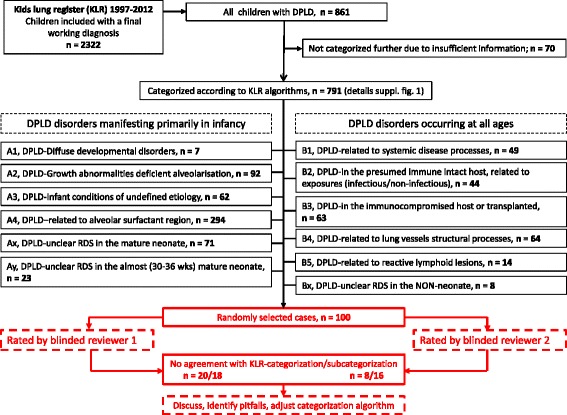
Overview on study design. The upper part (black) of the figure shows the patients collected in the kids lung register (KLR) and categorized according to the KLR algorithm between 1997 and 2012. Patients received a working diagnosis and were categorized into DPLD categories and subcategories; the latter process is described in more detail in Addditional file 2: Figure S1. The lower part of the figure (red) describes the workflow used for the re-categorization of 100 cases selected randomly and in proportion of their occurrence in the KLR. Two reviewers (AI, MG) re-assessed those cases blinded and independently and obtained a working diagnosis, categorization and sub-categorization according to the workflow in the lower part of Addditional file 2: Figure S1 (red)

For the cases referred to the KLR the categorization system for DPLD suggested by Deutsch et al. [1] was further developed; three additional categories were introduced (Additional file 1: Table S1) to accommodate cases with “unclear respiratory distress syndrome” in the mature neonate (Ax) and in the almost mature neonate (Ay) and “unclear respiratory distress syndrome” in the non-neonate (By). These categories allow the future analysis of unclear cases. In addition, the rather wide category “disorders masquerading as ILD” was dissolved into two more specific categories: “DPLD related to lung vessels structural processes” (B4) and “DPLD related to reactive lymphoid lesions” (B5) (Fig. 1).

Practical categorization rules were initially set up by the KLR (Table 1) to assure consistent categorization. 2322 children were referred to the KLR between 1997 and 2012. DPLD was suspected in a child with (1) respiratory symptoms and signs such as cough, tachy-/dyspnea at rest or with exercise, crackles, retractions, digital clubbing, failure to thrive, or respiratory failure, and (2) hypoxemia, and (3) diffuse radiological abnormalities and (4) if feasible and available, abnormalities in pulmonary function testing. Minimum duration of symptoms was 4 weeks.

**Table 1:** Rules for allocating a “final working diagnosis” to the disease categories and subcategories

General rules	Examples
1. A final working diagnosis is established based on the available information	
The final working diagnosis is the diagnosis with the highest likelihood. Even if some diagnostic tests are missing or the information level is low, a final working diagnosis is defined and used for categorization. Clinical symptoms (cough, dyspnea, etc.) are only considered informative for categorization if typical for the diagnosis	-Sarcoidosis is diagnosed based on chronic dyspnea, interstitial fine nodules, granulomatous skin lesions in biopsy, and increased angiotensin converting enzyme levels.
	-Respiratory distress in the mature neonate as DPLD is diagnosed after the exclusion of infectious, cardiac, metabolic, neurologic and localized pulmonary causes.
	-Tachypnoe in infants with NEHI
2. DPLD or not? Are any aspects of the final working diagnosis related to DPLD?	
(a) Yes: the case should be categorized in the DPLD system	-Child with juvenile myelomonocytic leukemia and dyspnea, cough and reversible airway obstruction. On CT scan no evidence for obliterative bronchiolitis and bronchiectasis.
(b) No: no categorization in the DPLD system	⇨Airway disease, no DPLD
	-Same history with same findings, but on CT scan septal thickening and centrilobular nodules
	⇨DPLD-in the immunocompromised host or transplanted (B3)
	-Pneumonia in a patient with chronic granulomatous disease, no evidence of an interstitial lung disease
	**⇨**systemic disease, no DPLD
3. Systemic or lung-only condition? Is the lung disease part of a systemic disease process or is it a lung-only condition?	
a) Allocation of a lung disease as part of a systemic disease process is preferred over classifying as lung-only DPLD, if there is any evidence for the involvement of systemic structures	-Clinical, BAL or histological evidence for pulmonary hemorrhage without any evidence for systemic involvement, diagnosis of idiopathic pulmonary hemosiderosis
*Reason*: it is more likely that the systemic disease causes a lung problem, than that an independent rare lung disease emerges in addition to a DPLD related to a systemic disease.	**⇨**DPLD - related to lung vessels structural processes (B4)
*Consequences*:	-Pulmonary hemorrhage and a disease-causing mutation for Osler’s disease
	**⇨**DPLD - related to systemic disease processes (B1)
(i) same histological pattern may be present in different categories	-Pulmonary hemorrhage and celiac disease
	**⇨**DPLD - related to systemic disease processes (B1)
(ii) carefully re-evaluate for potential lung disease only	
b) Allocation of a disease as a lung-only DPLD in the presence of hints for the involvement of systemic structures should only be done if convincing evidence supports a lung-only DPLD	-Acute lymphatic leukemia treated with chemotherapy and stem cell transplant, development of pulmonary pathology, histologically NSIP
	**⇨**DPLD – related to systemic disease processes (B1): lung injury as a complication or therapy more likely than independent lung disease
	-Same history, but detection of two disease causing ABCA3 mutations in the patient
	**⇨**DPLD – related to alveolar surfactant region (A4): lung-only DPLD in addition to oncologic disease
4. Select a category and a subcategory which best accommodates the final working diagnosis	
Prefer the category/subcategory with	
(a) a better causal link/explanation for the lung disease: cause of pulmonary disease is for example ranked higher than the histological pattern alone, since the same histological result can be allocated to several categories. If the cause is not determinable, the most likely association of the histological pattern with a disease is selected.	-a patient with a drug-induced hypersensitivity reaction of the lung and the histological pattern of NSIP
	**⇨**DPLD - in the presumed immune intact host, related to exposures (B2)
	-A patient with NSIP and no further clinical information
	**⇨**DPLD - related to the alveolar surfactant region (A4) is selected and awaits further molecular characterization, as NSIP can be associated with SFTPC, ABCA3, or TTF1/Nkx2.1 mutations
(b) the overall better proof, even if less specific	-BPD-cLDI is preferred over pulmonary hypoplasia as the latter can reliably only be assessed by radial counting of pathology specimens or experimentally by using novel imaging techniques not routinely available

Abbreviations: ABCA3, ATP-binding cassette sub-family A member 3, *BAL* bronchoalveolar lavage, *BPD-cLDI* bronchopulmonary dysplasia - chronic lung disease of infancy, *DPLD* diffuse parenchymal lung disease, *NEHI* Neuroendocrine cell hyperplasia of infancy, *NSIP* non-specific interstitial pneumonitis, *SFTPC* surfactant protein C, *TTF1* thyroid transcription factor 1

During capture of the cases with suspected DPLD the referring physician mostly specialized in pediatric pulmonology on the level of a tertiary or university hospital diagnosed the patients in cooperation with the radiologist and in cases with biopsy the pathologist. Available material included a clinical history, biochemical, radiological, histological and genetic data of varying level of detail. A diagnosis was independently also established by each of the KLR experts: F. B., a pathologist; P. L. a geneticist; M. G., a pediatric clinician and pulmonologist (Additional file 2: Figure S1).

A four-step algorithm was used for categorization (Table **1**): in a first step, a “final working diagnosis” was defined by consensus discussion, entered into the data-base and used for categorization and sub-categorization. In a second step it was analyzed if the final working diagnosis was related to DPLD. In a third step it was decided whether the patient suffered from a lung only condition or if the lung disease was part of a systemic disease. Of note, the latter are not restricted to the diagnosis of category B1. Other organ systems than the lungs may also be involved in diseases of categories B3, B4, B5, A1 and in particular A2 (Fig. 1, Additional file 1: Table S1). In a fourth step, the appropriate category and subcategory were selected, preferring the strongest causal explanation of the condition and the most conclusive supporting evidence taking into account the categorization rules shown in Table 1. A total of 791 children of 2322 children in the KLR qualified for childhood DPLD and were further categorized into one of the 12 DPLD categories (Fig. 1, Additional file 1: Table S1) and the respective subcategories.

### Workflow for re-rating of selected DPLD cases retrieved from the KLR

The validity and observer-dependency was tested in 2012 using a systematic sample of 100 DPLD cases from the cohort of 791 children previously categorized according to the KLR algorithm. 100 cases were selected randomly and in proportion to their frequency in the KLR categories. All 100 cases were pseudonymized for blinded and independent re-categorization by two pediatric pneumologists, familiar with the KLR categorization system (Fig. 1, red lowever part, Additional file 3: Tables S2, Additional file 4: Table S3). One of the physicians involved in re-rating was already involved in the initial categorization, the other was an independent physician. A final working diagnosis was newly established by each re-rater and allocated in the KLR categorization system, again according to the rules indicated in Table 1 and the workflow detailed in Additional file 2: Figure S1, red part. As re-rating of the categorization was the goal of the study the initial categorization obtained during routine work-flow was set as the correct one. The overall frequencies of diagnostics available for the establishment of the final working diagnosis, categorization and sub-categorization are indicated in Additional file 5: Table S4.

### Ethics Statement

All participants gave their written informed consent to participate in the KLR consultation and diagnosis program. The retrospective analysis of the data was approved by the institutional review board (EK 026–06). Prospective collection and analysis of data was approved in the GOLD.net project (EK 257–10), and analysis was performed under the project FP7-305653-chILD-EU (EK 111–13).

## Results

Of 2322 children referred to the KLR between 1997 and 2012, 861 were related to DPLD and included into this cohort. Subjects with insufficient information were excluded. 791 remaining subjects were assigned to the 12 DPLD categories (Fig. 1). Of these subjects 55 % were male; their age at presentation was 4.2 ± 5.5 years (median 1.0 years, range 0 to 20). 549 subjects had a lung disease manifesting primarily in infancy, of which the largest number (294) was assigned to the category A4 (DPLD–related to alveolar surfactant region) (Fig. 1).

Re-categorization of 100 cases was in agreement with the first allocation in more than 80 % (Table 2). Analysis of deviation in allocation showed that four kinds of allocation mistakes were made (Table 2): (1) one source for error was a lack of appreciation of all details available in the medical records; most of these mistakes were associated with wrong allocation of subcategories. (2) a second rater-related source of error was too little knowledge of and erroneous application of the categorization rules. For example, chromosomal abnormalities are listed as an independent category in A2 because they are present at birth and mainly manifest in infancy, and not in B1, i.e. related to systemic disease processes. (3) For some cases previously categorized, the re-rater decided that data was insufficient. In one case for instance, clinical and radiological data was available, however no genetic or histologic information. Information on low levels of hydrophobic surfactant protein content of BAL was not judged to be helpful (4).

**Table 2:** Results of blinded re-rating of 100 subjects with pediatric DPLD by two independent raters and reasons for incorrect rating (see individual values in Additional file 3: Table S2)

	Blinded rater 1		Blinded rater 2	
	Category	Subcategory	Category	Subcategory
Correct categorization	80	82	92	84
Non-correct categorization	20	18	8	16
1 Reports not appreciated/read in detail (= true mistake of rater)	5	8	2	6
2 Poor knowledge of the classification rules	3	4	2	4
3 Insufficient data on case	8	0	2	2
4 Deficit of the classification system	4	6	2	4

Data are absolute numbers (total *n* = 100) or %

Lastly, shortcomings of the categorization system itself lead to non-correct categorization: main deficits of the categorization system were observed for the differentiation of chronic tachypnea of infancy (A3), and for diseases involving the parenchyma but also or primarily the peripheral airways. The latter, such as post-infectious obliterative bronchiolitis and Mac-Leod-Swyer-James-Syndrome, both for immune-competent and immune-compromised hosts, were frequently categorized as airway disorders and not as DPLD. The former, i.e. infants with tachypnea were identified as neuroendocrine cell hyperplasia, even if there was no biopsy available. A comprehensive list of erroneous classification is displayed in Additional files 3 and 4: Tables S2, S3.

## Discussion

Here we describe an algorithm to categorize children with DPLD; we defined and assessed rules for categorization and suggest a tool for establishing large cohorts of consistently categorized subjects with rare pulmonary diseases. We thus provide an important basis for the development of consensus-based, international guidelines for categorization and management of pediatric DPLD. Consistent categorization is indispensable for handling individual cases in registries and biobanks appropriately. It allows to combine or split diagnosis groups and to compare subcategories and categories. A consistent categorization system is the basis for future adjustments, such as the inclusion of new molecular disease entities or of novel diagnostic methods. A specific working diagnosis may change over time or knowledge may evolve on a particular subject, however, the allocation rules should not change, representing an important constant term.

In this study, several important barriers to a consistent categorization of rare lung diseases were identified. It was shown that consistent categorization needs to be repeatedly practiced especially for the use in large registers. Continuous evaluation of the categorization process within a register will be an important element of quality control.

Lack of sufficient data in a case is a common problem in clinical practice, hindering the establishment of a correct diagnosis. Data may be insufficient for many reasons, such as high costs for diagnostic testing, invasiveness of tests (e.g. lung biopsy), missing data or poor quality of the data (e.g. incomplete history, CT scans performed in infants with improper technique). The problem of insufficient data should not distract from making a diagnosis. Appreciating all information and details available will yield a final working diagnosis, which should be clearly indicated. Even if the diagnosis leaves open questions e.g. “unclear RDS in the mature neonate”, these cases must nevertheless be categorized. For this purpose the categories Ax, Ay, Bx were created (Fig. 1, Additional file 1: Table S1). The cases in these categories can (and must) be systematically revisited and if more information becomes available, should be allocated into more specific categories. These patients can furthermore be included into non-hypothesis based screening projects, like exome sequencing or disease marker identification projects, with the aim to identify previously unknown disease causes or to determine disease activity.

Any classification system is continuously evolving. Increasing knowledge on molecular disease mechanisms allows the definition of new entities, which must be easily accommodated in the categorization system, as is the case for the current system.

It is furthermore essential to continuously take note of potential areas of uncertainty within the system and to clarify these: there are for example entities for which no precise diagnostic criteria are available, such as the differentiation of infants with chronic tachypnea in the absence of a lung biopsy (See examples in Additional file 3: Table S2). Another area which needs clarification is the categorization of diffuse parenchymal diseases which also involve the distal airways. These patients overlap with those presenting primarily as obstructive airway diseases, but cannot merely be classified as such, because the remodeling of the lung tissue component is dominant. A precise definition of all subcategories is not yet available but will be significant to be developed as “gold-standard”.

Lastly, a rating of the confidence level of the quality of the data used for establishing the working diagnosis for the individual cases would be a valuable additional indicator and is desirable to be established in the future.

Using a clinically oriented categorization system such as the one presented here has the advantage that different registries or studies using the same definitions and rules may be compared or combined for analysis. Consistent application of a clinically oriented categorization system is prerequisite for the establishment of urgently needed larger cohorts of patients with rare pediatric lung diseases.

## Conclusions

We present hands-on rules for the categorization of all pediatric DPLD, independent of the presence or absence of a lung biopsy or the quality of diagnostic data. We empirically identify pitfalls of categorization and suggest solutions for improvement with the aim to provide the basis for the development of consensus-based, international guidelines for the categorization and management of pediatric DPLD.

## Additional files

Additional file 1: Table S1. Definitions of diffuse parenchymal lung diseases (DPLD) - disease categories and subcategories. (DOCX 26 kb) Additional file 2: Figure S1. Routine work-flow used in the Kids lung register (KLR) to obtain a final working diagnosis and to categorize and subcategorize cases with suspected DPLD (black and red). The work-flow used for re-rating is depicted in red. *reference pathologist was Frank Brasch, **genetical diagnosis was made by Peter Lohse and ***lavage report on surfactant protein analysis, as well as the establishment of the final working diagnosis during routine KLR workflow were done by Matthias Griese. (PDF 176 kb) Additional file 3: Table S2. Blinded and independent re-rating of a random sample of 100 DPLD cases from the kids-lung register. Only differences to the original categorization are indicated; empty cells indicate correct categorization. (DOCX 35 kb) Additional file 4: Table S3. Summary of reasons for incorrect categorization. (DOCX 15 kb) Additional file 5: Table S4. Overall frequencies of diagnostics available for the establishment of the final working diagnosis, categorization and sub-categorization. Note that not all tests were performed in all patients. (DOCX 15 kb) Additional file 6: Table S5. Centers and physicians contributing cases to the kids-lung-register. (DOCX 34 kb)

## Abbreviations

DPLD: Diffuse parenchymal lung disease
KLR: Kids-lung-register
RDS: Respiratory distress syndrome

## References

[1] Deutsch GH, Young LR, Deterding RR, Fan LL, Dell SD, Bean JA, Brody AS, Nogee LM, Trapnell BC, Langston C, Pathology Cooperative G, Albright EA, Askin FB, Baker P, Chou PM, Cool CM, Coventry SC, Cutz E, Davis MM, Dishop MK, Galambos C, Patterson K, Travis WD, Wert SE, White FV (2007). Ch ILDRC-o: Diffuse lung disease in young children: application of a novel classification scheme. Am J Respir Crit Care Med.

[2] Griese M, Haug M, Brasch F, Freihorst A, Lohse P, von Kries R, Zimmermann T, Hartl D (2009). Incidence and classification of pediatric diffuse parenchymal lung diseases in Germany. Orphanet J Rare Dis.

[3] Dinwiddie R, Sharief N, Crawford O (2002). Idiopathic interstitial pneumonitis in children: a national survey in the United Kingdom and Ireland. Pediatr Pulmonol.

[4] Kurland G, Deterding RR, Hagood JS, Young LR, Brody AS, Castile RG, Dell S, Fan LL, Hamvas A, Hilman BC, Langston C, Nogee LM, Redding GJ (2013). American Thoracic Society Committee on Childhood Interstitial Lung D, the ch ILDRN: An official American Thoracic Society clinical practice guideline: classification, evaluation, and management of childhood interstitial lung disease in infancy. Am J Respir Crit Care Med.

[5] Clement A, Force ERST (2004). Task force on chronic interstitial lung disease in immunocompetent children. Eur Respir J.

[6] Rice A, Tran-Dang MA, Bush A, Nicholson AG (2013). Diffuse lung disease in infancy and childhood: expanding the chILD classification. Histopathology.

[7] Langston C, Dishop MK (2009). Diffuse lung disease in infancy: a proposed classification applied to 259 diagnostic biopsies. Pediatr Dev Pathol.

[8] Soares JJ, Deutsch GH, Moore PE, Fazili MF, Austin ED, Brown RF, Sokolow AG, Hilmes MA, Young LR (2013). Childhood interstitial lung diseases: an 18-year retrospective analysis. Pediatrics.

[9] The diagnosis, assessment and treatment of diffuse parenchymal lung disease in adults. Introduction. Thorax 1999, 54 Suppl 1:S1-14. 10.1136/thx.54.suppl_1.s1 PMC1765921 11006787

