# Supplementary material for: Categorizing diffuse parenchymal lung disease in children
Source: Orphanet J Rare Dis. 2015 Sep 25;10:122. doi: 10.1186/s13023-015-0339-1 (PMC4582630; doi:10.1186/s13023-015-0339-1)
Supplement: Additional file 1: Table S1. — Definitions of diffuse parenchymal lung diseases (DPLD) - disease categories and subcategories. (DOCX 26 kb) [file 13023_2015_339_MOESM1_ESM.docx]

Supplemental Table 1 Definitions of diffuse parenchymal lung diseases (DPLD) - disease categories and subcategories

| **Category** | | **Definition** | **Subcategories** |
| --- | --- | --- | --- |
| **A** | **DPLD disorders manifesting primarily in infancy** |  |  |
| A1 | Diffuse developmental disorders | Disorders of lung development leading to bilateral structurally abnormal lung parenchyma | - Acinar dysplasia  - Congenital alveolar dysplasia  - Alveolar-capillary dysplasia with pulmonary vein misalignment  - Alveolar-capillary dysplasia without misalignment of the vessels |
| A2 | Alveolarisation deficiencies | Disorders with deficient alveolarisation due to pre- or postnatal abnormal lung growth | - Pulmonary hypoplasia, primary (idiopathic)  - Pulmonary hypoplasia, secondary (e.g. diaphragmatic hernia)  - Related to chromosomal disorders  - Related to congenital heart disease (without chromosomal disorder)  - Bronchopulmonary dysplasia - chronic lung disease of prematurity (BPD-cLDI)  -Wilson Mikity syndrome |
| A3 | Specific conditions of undefined etiology | Disorders in infants with chronic tachypnea without a yet recognized genetic disorder, with a clear histological and/or radiological pattern | - Chronic tachypnea of infancy (CTI)  - Neuroendocrine cell hyperplasia of infancy (NEHI)  - Pulmonary interstitial glycogenosis |
| A4 | Surfactant dysfunction disorders | Disorders related to the alveolar surfactant region, with a proven genetic defect or with a defined histological pattern | - ABCA3 mutations  - SFTPC mutations  - SFTPB mutations  - NKX2-1 mutations  - GMCSF-RA mutations  - GMCSF-RB mutations  - Chronic pneumonitis of infancy (CPI)  - Usual interstitial pneumonia (UIP)  - Nonspecific interstitial pneumonia (NSIP)  - Desquamative interstitial pneumonia (DIP)  - Pulmonary alveolar proteinosis (PAP) with GMCSF autoantibodies  - Pulmonary alveolar proteinosis (PAP) without GMCSF autoantibodies  - Pulmonary alveolar proteinosis (PAP) juvenile  - Pulmonary alveolar proteinosis (PAP) neonatal  - Pulmonary alveolar proteinosis (PAP) secondary to associated disease  - Pulmonary alveolar proteinosis (PAP) with GATA2 mutation  - Other surfactant-associated disorders without a yet defined genetic defect  - Alveolar microlithiasis  - Lipoid pneumonitis, Cholesterol pneumonia |
| Ax | Unclear Respiratory Distress Syndrome in the mature neonate | DPLD disorders in neonates of gestational age > 37 weeks, after the exclusion of neonatal infection, congenital heart disease, inborn metabolic diseases, central or peripheral nervous system disorders | - Familial  - Pulmonary hypertension  - No or very low surfactant proteins SP-B or SP-C biochemically |
| Ay | Unclear Respiratory Distress Syndrome in the almost (30-36 weeks) mature neonate | DPLD disorders in neonates of gestational age 30 to 36 weeks, unlikely due to immaturity alone, after the exclusion of neonatal infection, congenital heart disease, inborn metabolic diseases, central or peripheral nervous system disorders | - Familial  - Pulmonary hypertension  - No or very low surfactant proteins SP-B or SP-C biochemically |
| **B** | **DPLD disorders occurring at all ages** |  |  |
| B1 | DPLD related to systemic disease processes | DPLD due to systemic disorders with pulmonary manifestation | - Achondroplasia, e.g. cartilage-hair hypoplasia  - Alagille syndrome  - Goodpasture’s disease  - Antisynthetase syndrome  - Behcet’s disease  - Birt-Hogg-Dubé syndrome  - Blau syndrome  - Congenital central hypoventilation syndrome (CCHS)  - Churg-Strauss syndrome (CSS)  - Congenital muscle disease  - Diffuse alveolar hemorrhage due to vasculitis disorder  - Disseminated Visceral Giant Cell Angiitis  - Erdheim-Chester disease  - Familial dysautonomia (FD)  - Giant cell arteritis  - Hermansky-Pudlak syndrome (HPS)  - Hoyeraal-Hreidarsson syndrome  - Hypercalcuric hypercalcemia  - Immune-mediated/collagen vascular disorders  - Lymphangioleiomyomatosis (LAM)  - Langerhans cell histiocytosis  - Osler’s disease  - Malignant infiltrates  - Microscopic polyangiitis  - Necrotizing sarcoid granulomatosis  - Neurofibromatosis, von Recklingshausen’s disease  - Other rare causes of granulomatous arteritis  - Polyarteritis nodosa  - Rubinstein-Taybi-syndrome  - Sarcoidosis  - Sickle cell disease  - Sinus histiocytosis with massive lymphadenopathy  - Stevens-Johnson syndrome - drug induced, with or without obliterative bronchiolitis  - Stevens-Johnson syndrome – idiopathic, with or without obliterative bronchiolitis  - Stevens-Johnson syndrome - infection related, with or without obliterative bronchiolitis  - Storage diseases  - Systemic lupus erythematosus  - Takayasu's arteritis  - Wegener’s granulomatosis |
| B2 | DPLD in the presumed immune- intact host, related to exposures (infectious/non-infectious) | DPLD as consequence of exposure to substances or infectious agents in the lungs in the immune-intact host | - Acute fibrinous and organizing pneumonia  - Aspiration syndromes, with or without obliterative bronchiolitis  - Diffuse alveolar damage and acute interstitial pneumonia  - Drug reactions  - Eosinophilic pneumonia  - Exogen allergic alveolitis, hypersensitivity pneumonitis  - Infectious/post-infectious processes, with or without bronchiolitis obliterans  - Occupational lung diseases and pneumoconioses  - Organizing pneumonia, cryptogenic (COP, former idiopathic BOOP) or secondary (OP)  - Radiation lung injury  - Respiratory bronchiolitis interstitial lung disease (RB-ILD)  - Swyer-James-syndrome  - Toxic inhalation, with or without obliterative bronchiolitis |
| B3 | DPLD in the immunocompro-mised or transplanted host | DPLD characteristic for and developing only in the presence of specific immune dysfunctions or transplantation-related immune processes | - Diffuse lung damage of unknown etiology  - Infections, due to antibody deficiencies  - Infections, due to phagocyte defects  - Infections, due to T cell deficiencies  - Infections, miscellaneous  - Nonspecific interstitial pneumonia (NSIP)  - Related to therapeutic intervention, with or without obliterative bronchiolitis  - Related to transplantation and rejection, with or without obliterative bronchiolitis |
| B4 | DPLD related to lung vessels structural processes | DPLD disorders due to dysfunctional structural components of the pulmonary veins, arteries, capillaries or lymphatics. Involvement of systemic or other organ vessels is absent or marginal. | - Pulmonary hypertension, primary or secondary  - Congestive changes related to cardiac dysfunction  - Idiopathic pulmonary hemosiderosis (IPH)  - Lymphatic disorders  - Pulmonary capillary hemangiomatosis  - Pulmonary hemorrhage, idiopathic or due to infection  - Veno-occlusive disease |
| B5 | DPLD related to reactive lymphoid lesions | DPLD due to non-malignant disorders affecting lymphocyte numbers and functions in the lungs | - Follicular bronchitis/bronchiolits  - Giant lymph node hyperplasia (Castleman´s disease)  - Intrapulmonary lymph nodes  - Lymphocytic interstitial pneumonia (LIP)  - Nodular lymphoid hyperplasia of the lung |
| Bx | Unclear Respiratory Distress Syndrome in the non-neonate | DPLD disorders in children older than 30 days, after the exclusion of infection, heart disease or circulatory disorders, metabolic diseases, central or peripheral nervous system disorders | - familial |
