# Supplementary material for: Categorizing diffuse parenchymal lung disease in children
Source: Orphanet J Rare Dis. 2015 Sep 25;10:122. doi: 10.1186/s13023-015-0339-1 (PMC4582630; doi:10.1186/s13023-015-0339-1)
Supplement: Additional file 2: Figure S1. — Routine work-flow used in the Kids lung register (KLR) to obtain a final working diagnosis and to categorize and subcategorize cases with suspected DPLD (black and red). The work-flow used for re-rating is depicted in red. *reference pathologist was Frank Brasch, **genetical diagnosis was made by Peter Lohse and ***lavage report on surfactant protein analysis, as well as the establishment of the final working diagnosis during routine KLR workflow were done by Matthias Griese. (PDF 176 kb) [file 13023_2015_339_MOESM2_ESM.pdf]

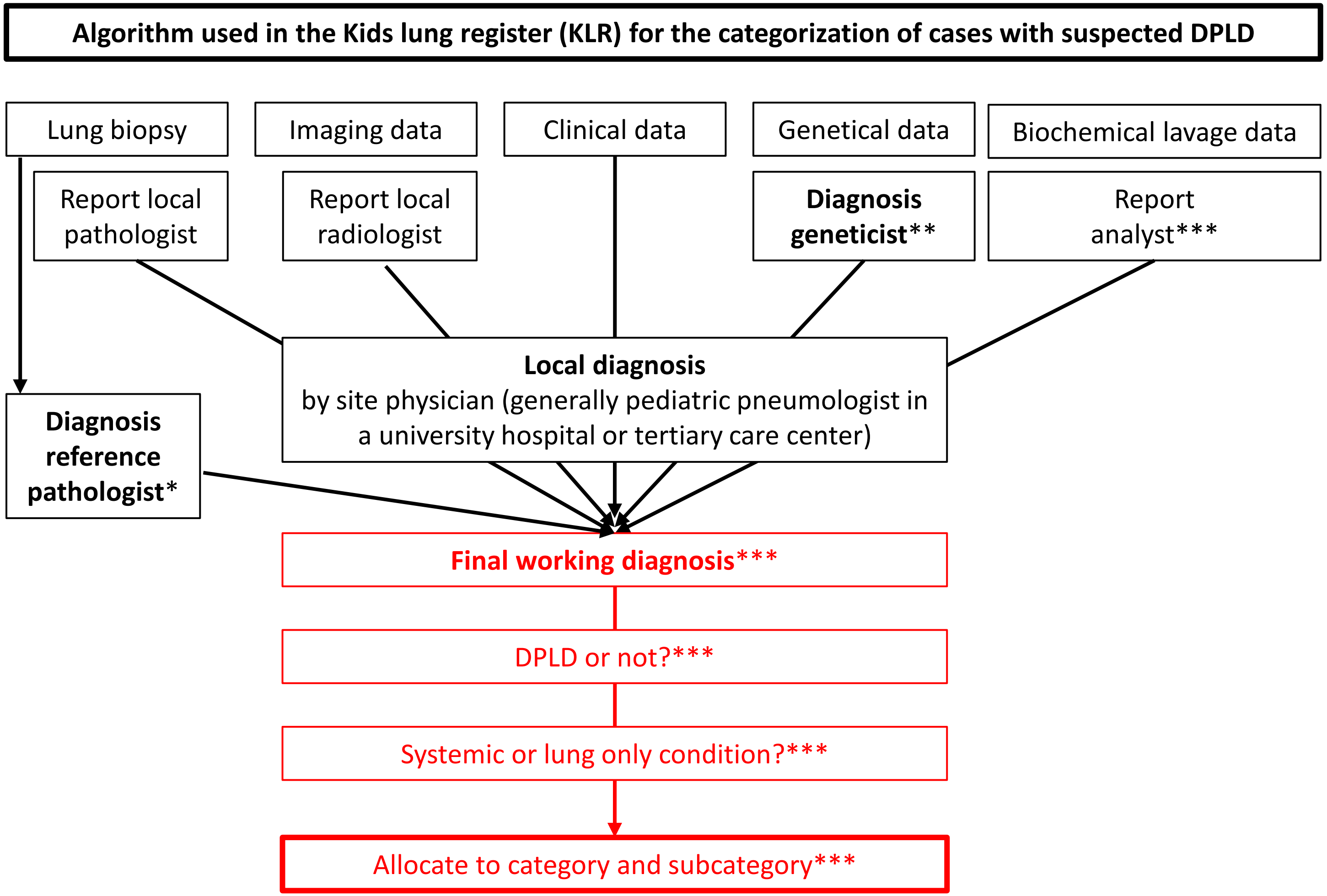

Routine work-flow used in the Kids lung register (KLR) to obtain a final working diagnosis and to categorize and subcategorize cases with suspected DPLD (black and red). The work-flow used for re-rating is depicted in red.

*reference pathologist was Frank Brasch, **genetical diagnosis was made by Peter Lohse and ***lavage report on surfactant protein analysis, as well as the establishment of the final working diagnosis during routine KLR workflow were done by Matthias Griese
