# Supplementary material for: Categorizing diffuse parenchymal lung disease in children
Source: Orphanet J Rare Dis. 2015 Sep 25;10:122. doi: 10.1186/s13023-015-0339-1 (PMC4582630; doi:10.1186/s13023-015-0339-1)
Supplement: Additional file 3: Table S2. — Blinded and independent re-rating of a random sample of 100 DPLD cases from the kids-lung register. Only differences to the original categorization are indicated; empty cells indicate correct categorization. (DOCX 35 kb) [file 13023_2015_339_MOESM3_ESM.docx]

Suppl. Table 2. Blinded and independent re-rating of a random sample of 100 DPLD cases from the kids-lung register. Only differences to the original categorization are indicated; empty cells indicate correct categorization.

|  |  | **Kids-lung register (KLR) diagnoses and categorization** | | **Blinded rater 1** | | | | **Blinded rater 2** | | | |
| --- | --- | --- | --- | --- | --- | --- | --- | --- | --- | --- | --- |
| **ID** | **KLR-ID** | **Category** | **Subcategory** | **Category** | **Reason for Error*** | **Subcategory** | **Reason for Error*** | **Category** | **Reason for Error*** | **Subcategory** | **Reason for Error*** |
| 1 | 254 | A1 | Alveolo capillary dysplasia |  |  |  |  |  |  |  |  |
| 2 | 202 | A1 | Congenital alveolar dysplasia | A4 | 1 | Diffuse Alveolar Damage and Acute Interstitial Pneumonia | 1 |  |  |  |  |
| 3 | 1863 | A2 | Pulmonary hypoplasia |  |  |  |  |  |  |  |  |
| 4 | 818 | A2 | Pulmonary hypoplasia | D | 1 | Congenital Bronchial Cartilage Deficiency | 1 |  |  |  |  |
| 5 | 422 | A2 | Pulmonary hypoplasia |  |  | Related to preterm birth (BPD-cLDI) | 2b |  |  |  |  |
| 6 | 1400 | A2 | Related to chromosomal disorders | B1 | 2a |  |  |  |  |  |  |
| 7 | 491 | A2 | Related to preterm birth (BPD-cLDI) |  |  |  |  |  |  | Related to preterm birth (Wilson Mikity, new BPD) | 4c |
| 8 | 225 | A2 | Related to preterm birth (BPD-cLDI) |  |  |  |  |  |  |  |  |
| 9 | 1448 | A2 | Related to preterm birth (BPD-cLDI) |  |  |  |  |  |  |  |  |
| 10 | 1891 | A2 | Related to preterm birth (BPD-cLDI) |  |  |  |  |  |  |  |  |
| 11 | 1404 | A2 | Related to preterm birth (Wilson Mikity, new BPD) | A4 | 2b | Diffuse Alveolar Damage and Acute Interstitial Pneumonia | 2b |  |  | Related to preterm birth (BPD-cLDI) | 4c |
| 12 | 600 | A2 | Related to preterm birth (Wilson Mikity, new BPD) |  | 4a | Related to preterm birth (BPD-cLDI) | 4c |  |  |  |  |
| 13 | 544 | A3 | Chronic tachypnoe of infancy (CTI) | D | 4a | Bronchiolitis obliterans | 4a |  |  |  |  |
| 14 | 697 | A3 | Chronic tachypnoe of infancy (CTI) | B2 |  | Infectious/post-infectious processes | 4a |  |  |  |  |
| 15 | 1735 | A3 | Chronic tachypnoe of infancy (CTI) |  |  |  |  | D | 4a | Bronchitis, chron. neutrophil (BAL) | 4a |
| 16 | 2469 | A3 | Chronic tachypnoe of infancy (CTI) |  |  | Neuroendocrine cell hyperplasia of infancy | 1 |  |  |  |  |
| 17 | 1641 | A3 | Chronic tachypnoe of infancy (CTI) |  |  |  |  |  |  |  |  |
| 18 | 2231 | A3 | Chronic tachypnoe of infancy (CTI) |  |  |  |  |  |  |  |  |
| 19 | 1610 | A3 | Chronic tachypnoe of infancy (CTI) |  |  |  |  |  |  |  |  |
| 20 | 1909 | A3 | Chronic tachypnoe of infancy (CTI) |  |  |  |  |  |  |  |  |
| 21 | 2163 | A3 | Neuroendocrine cell hyperplasia of infancy |  |  | Chronic tachypnoe of infancy (CTI) | 1 |  |  |  |  |
| 22 | 1344 | A3 | Neuroendocrine cell hyperplasia of infancy |  |  |  |  |  |  |  |  |
| 23 | 687 | A3 | Neuroendocrine cell hyperplasia of infancy |  |  |  |  |  |  |  |  |
| 24 | 1703 | A3 | Neuroendocrine cell hyperplasia of infancy |  |  |  |  |  |  |  |  |
| 25 | 2152 | A3 | Neuroendocrine cell hyperplasia of infancy |  |  |  |  |  |  |  |  |
| 26 | 2316 | A3 | Neuroendocrine cell hyperplasia of infancy |  |  |  |  |  |  |  |  |
| 27 | 1831 | A3 | Pulmonary interstitial glycogenosis (PIG) |  |  |  |  |  |  |  |  |
| 28 | 627 | A4 | ABCA3 mutations 1 |  |  |  |  |  |  | NSIP, cellular | 2d |
| 29 | 208 | A4 | ABCA3 mutations 2 |  |  |  |  |  |  |  |  |
| 30 | 458 | A4 | ABCA3 mutations 2 |  |  |  |  |  |  |  |  |
| 31 | 2202 | A4 | ABCA3 mutations 2 |  |  |  |  |  |  |  |  |
| 32 | 1616 | A4 | Alveolar microlithiasis |  |  |  |  |  |  |  |  |
| 33 | 1900 | A4 | Chronic pneumonitis of infancy (CPI) |  |  | PAP, neonatal | 1 |  |  |  |  |
| 34 | 1916 | A4 | DIP | Insuff. data | 3 |  |  | Insuff. data | 3 |  |  |
| 35 | 251 | A4 | DIP |  |  |  |  | B1 | 2c | Drug reaction | 2b |
| 36 | 68 | A4 | DIP |  |  | PAP, sec. to associated disease | 1 |  |  |  |  |
| 37 | 1859 | A4 | Lipoidpneumonitis, Cholesterol pneumonia |  |  |  |  |  |  |  |  |
| 38 | 227 | A4 | Lipoidpneumonitis, Cholesterol pneumonia |  |  |  |  |  |  |  |  |
| 39 | 1399 | A4 | Nkx21 gene defect |  |  |  |  |  |  |  |  |
| 40 | 507 | A4 | Nonspecific interstitial pneumonia (NSIP) |  |  |  |  |  |  | NSIP, cellular | 1 |
| 41 | 2229 | A4 | Nonspecific interstitial pneumonia (NSIP) |  |  |  |  |  |  |  |  |
| 42 | 2066 | A4 | Nonspecific interstitial pneumonia (NSIP) |  |  |  |  |  |  |  |  |
| 43 | 2173 | A4 | Nonspecific interstitial pneumonia (NSIP) |  |  |  |  |  |  |  |  |
| 44 | 173 | A4 | PAP, adult NO GMCSF autoantibodies |  |  |  |  |  |  |  |  |
| 45 | 143 | A4 | PAP, adult with GMCSF autoantibodies |  |  |  |  |  |  |  |  |
| 46 | 163 | A4 | PAP, GATA2 mutation |  |  |  |  |  |  |  |  |
| 47 | 195 | A4 | PAP, GM-CSF-RA Mutation |  |  |  |  |  |  |  |  |
| 48 | 1787 | A4 | PAP, GM-CSF-RA Mutation |  |  |  |  |  |  |  |  |
| 49 | 629 | A4 | Surfactant protein B mutations |  |  |  |  |  |  |  |  |
| 50 | 336 | A4 | Surfactant protein C mutations |  |  |  |  |  |  |  |  |
| 51 | 240 | A4 | Surfactant protein C mutations |  |  |  |  |  |  |  |  |
| 52 | 1446 | A4 | Surfactant protein C mutations |  |  |  |  |  |  |  |  |
| 53 | 2064 | A4 | Surfactant protein C mutations |  |  |  |  |  |  |  |  |
| 54 | 599 | Ax | Insuff. data |  |  |  |  |  |  |  |  |
| 55 | 717 | Ax | No or very low SP-C biochemically | Insuff. data | 3 |  |  |  |  | Insuff. data | 3 |
| 56 | 713 | Ax | Pulmonary hypertension |  |  |  |  |  |  | Insuff. data | 3 |
| 57 | 471 | Ay | Insuff. data | Insuff. data | 3 |  |  |  |  |  |  |
| 58 | 468 | Ay | No or very low SP-C biochemically | Insuff. data | 3 |  |  |  |  |  |  |
| 59 | 551 | Ay | No or very low SP-C biochemically | A2 | 1 | Related to preterm birth (BPD-cLDI) | 1 |  |  |  |  |
| 60 | 2073 | B1 | Hermansky-Pudlak Syndrome |  |  |  |  |  |  |  |  |
| 61 | 1509 | B1 | Immune-mediated/collagen vascular disorders |  |  | Diffuse alveolar hemorrhage due to vasculitic disorders | 4c | B2 | 1 | Related to therapeutic intervention | 1 |
| 62 | 1795 | B1 | Langerhans cell histiocytosis |  |  |  |  |  |  |  |  |
| 63 | 2479 | B1 | M. Osler |  |  |  |  |  |  |  |  |
| 64 | 1649 | B1 | Rubinstein-Taybi-syndrome |  |  |  |  | A1 | 2a | Related to chromosomal disorders | 2a |
| 65 | 1343 | B1 | Sarcoidosis | Insuff. data | 3 |  |  |  |  |  |  |
| 66 | 1934 | B1 | Sarcoidosis |  |  |  |  |  |  |  |  |
| 67 | 1983 | B1 | Storage diseases | A4 | 2c | PAP, neonatal | 2c |  |  |  |  |
| 68 | 284 | B1 | Storage diseases |  |  |  |  |  |  |  |  |
| 69 | 1579 | B1 | Wegener Granulomatosis |  |  |  |  |  |  |  |  |
| 70 | 692 | B2 | Aspiration syndromes | Insuff. data | 3 |  |  |  |  |  |  |
| 71 | 1688 | B2 | Eosinophilic pneumonitis |  |  |  |  | B1 | 1 | Churg-Strauss Syndrome | 1 |
| 72 | 233 | B2 | Eosinophilic pneumonitis |  |  |  |  |  |  |  |  |
| 73 | 264 | B2 | Exogen allergic alveolitis |  |  |  |  |  |  |  |  |
| 74 | 644 | B2 | Exogen allergic alveolitis |  |  |  |  |  |  |  |  |
| 75 | 1453 | B2 | Exogen allergic alveolitis |  |  |  |  |  |  |  |  |
| 76 | 1638 | B2 | Exogen allergic alveolitis |  |  |  |  |  |  |  |  |
| 77 | 1627 | B2 | Mac-Leod-Swyer-James-Syndrome | D | 4b | Bronchiolitis obliterans | 4b |  |  |  |  |
| 78 | 1920 | B3 | Diffuse lung damage of unknown etiology | Insuff. data | 1 |  |  | Insuff. data | 1 |  |  |
| 79 | 535 | B3 | Infections–Antibody deficiencies | D | 4d | Infectious Bronchiolitis and Postinfectious Constrictive Bronchiolitis | 4d |  |  |  |  |
| 80 | 1696 | B3 | Infections–Antibody deficiencies |  |  |  |  | D | 4d | chronic Bronchitis | 4d |
| 81 | 721 | B3 | Infections–Antibody deficiencies |  |  |  |  |  |  |  |  |
| 82 | 1329 | B3 | Infections–T cell deficiencies |  |  |  |  |  |  | Infections–Miscellaneous | 1 |
| 83 | 678 | B3 | Related to therapeutic intervention |  |  |  |  |  |  |  |  |
| 84 | 218 | B3 | Related to transplantation and rejection | Insuff. data | 3 |  |  |  |  |  |  |
| 85 | 1276 | B3 | Related to transplantation and rejection |  |  |  |  |  |  |  |  |
| 86 | 1577 | B3 | Related to transplantation and rejection |  |  |  |  |  |  |  |  |
| 87 | 1620 | B3 | Related to transplantation and rejection |  |  |  |  |  |  |  |  |
| 88 | 1960 | B4 | Idiopathic pulmonary hemosiderosis |  |  | Pulmonary hemorrhage | 2c |  |  |  |  |
| 89 | 1551 | B4 | Idiopathic pulmonary hemosiderosis |  |  |  |  |  |  |  |  |
| 90 | 1816 | B4 | Idiopathic pulmonary hemosiderosis |  |  |  |  |  |  |  |  |
| 91 | 1988 | B4 | Primary pulmonary hypertension due to Chr 2q33 |  |  |  |  |  |  | Pulmonary hypertension | 2d |
| 92 | 1894 | B4 | Pulmonary capillary hemangiomatosis |  |  |  |  |  |  |  |  |
| 93 | 1419 | B4 | Pulmonary capillary hemangiomatosis |  |  |  |  |  |  |  |  |
| 94 | 306 | B4 | Pulmonary capillary hemangiomatosis |  |  |  |  |  |  |  |  |
| 95 | 1665 | B4 | Pulmonary hypertension |  |  |  |  |  |  |  |  |
| 96 | 541 | B5 | Lymphocytic interstitial pneumonia (LIP) | B1 | 1 | Immune-mediated/collagen vascular disorders | 1 |  |  |  |  |
| 97 | 346 | B5 | Lymphocytic interstitial pneumonia (LIP) |  |  |  |  |  |  | Follicular bronchiolitis | 1 |
| 98 | 1679 | B5 | Lymphocytic interstitial pneumonia (LIP) |  |  |  |  |  |  |  |  |
| 99 | 732 | D | cardiac cause | Ay | 1 |  |  |  |  | Bronchiolitis obliterans | 1 |
| 100 | 1494 | F | Pneumonia from Pneumocystis |  |  |  |  |  |  |  |  |

*Reason for Error:

1 Reports not appreciated/read in detail (= true mistake of physician), 2 Poor knowledge of the classification rules, 3 Insufficient data on case, 4 Deficit of the classification system
