# Supplementary material for: Categorizing diffuse parenchymal lung disease in children
Source: Orphanet J Rare Dis. 2015 Sep 25;10:122. doi: 10.1186/s13023-015-0339-1 (PMC4582630; doi:10.1186/s13023-015-0339-1)
Supplement: Additional file 4: Table S3. — Summary of reasons for incorrect categorization. (DOCX 15 kb) [file 13023_2015_339_MOESM4_ESM.docx]

Supplemental Table 3. Summary of reasons for incorrect categorization

|  | **Blinded rater 1** | | | | **Blinded rater 2** | | | |  |
| --- | --- | --- | --- | --- | --- | --- | --- | --- | --- |
|  | **Category** | **Subcategory** | | | **Category** | | **Subcategory** | |  |
| **% non-correct categorizations** | **20** | **18** | | | **8** | | **16** | | |
| 1 Reports not appreciated/read in detail (= true mistake of physician) | 5 | 8 | | | 2 | | 6 | | |
| 2 Poor knowledge of classification rules | 3 | 4 | | | 2 | | 4 | | |
| *Correct application of rules* |  |  | | |  | |  | | |
| *2a Related to chromosomal abnormality is A2, not B1* | *1* | *0* | | | *1* | | *1* | | |
| *2b Choose more likely, although less specific classification* | *1* | *2* | | | *0* | | *1* | | |
| *2c Select systemic disorder if there is any involvement of systemic structures instead of local pulmonary alone* | *1* | *2* | | | *1* | | *0* | | |
| *2d Molecular result to be rated higher than histologic result* | *0* | | *0* | *0* | | *2* | |  |  |
| 3 Insufficient data on case | 8 | | 0 | 2 | | 2 | |  |  |
| 4 Deficit of the classification | 4 | | 6 | 2 | | 4 | |  |  |
| *4a Differentiation of NEHI* | *2* | | *2* | *1* | | *1* | |  |  |
| *4b Post infectious BO can also be classified as airway disease and if one-sided as Mac-Leod-Swyer-James-Syndrome* | *1* | | *1* | *0* | | *0* | |  |  |
| *4c No precise diagnostic criteria for an entity available* | *0* | | *2* | *0* | | *2* | |  |  |
| *4d DPLD in the immunocompromised host/transplanted needs differentiation from airway disease or obstructive bronchiolitis or bronchitis* | *1* | | *1* | *1* | | *1* | |  |  |
