# Supplementary material for: Categorizing diffuse parenchymal lung disease in children
Source: Orphanet J Rare Dis. 2015 Sep 25;10:122. doi: 10.1186/s13023-015-0339-1 (PMC4582630; doi:10.1186/s13023-015-0339-1)
Supplement: Additional file 5: Table S4. — Overall frequencies of diagnostics available for the establishment of the final working diagnosis, categorization and sub-categorization. Note that not all tests were performed in all patients. (DOCX 15 kb) [file 13023_2015_339_MOESM5_ESM.docx]

Supplemental Table 4. Overall frequencies of diagnostics available for the establishment of the final working diagnosis, categorization and sub-categorization. Note that not all tests were performed in all patients.

|  | Category | N | Molecular genetics done | Chest CT done | Lung biopsy done | Lavage surfactant analysis done |
| --- | --- | --- | --- | --- | --- | --- |
| A1 | Diffuse developmental disorders | 2 | 2 | 1 | 2 | 2 |
| A2 | Alveolarisation deficiencies | 10 | 7 | 6 | 4 | 7 |
| A3 | Specific conditions of undefined etiology | 15 | 9 | 14 | 10 | 7 |
| A4 | Surfactant dysfunction disorders | 26 | 21 | 20 | 16 | 16 |
| Ax | Unclear Respiratory Distress Syndrome in the mature neonate | 3 | 2 | 0 | 1 | 1 |
| Ay | Unclear Respiratory Distress Syndrome in the almost (30-36 weeks) mature neonate | 3 | 2 | 1 | 2 | 2 |
| B1 | DPLD related to systemic disease processes | 10 | 3 | 6 | 4 | 3 |
| B2 | DPLD in the presumed immune- intact host, related to exposures (infectious/non-infectious) | 8 | 2 | 6 | 6 | 3 |
| B3 | DPLD in the immunocompro-mised or transplanted host | 10 | 1 | 4 | 1 | 2 |
| B4 | DPLD related to lung vessels structural processes | 8 | 3 | 3 | 5 | 2 |
| B5 | DPLD related to reactive lymphoid lesions | 3 | 3 | 4 | 3 | 2 |
| D | Airway disorders | 1 | 0 | 1 | 0 | 1 |
| F | Lung infections | 1 | 0 | 1 | 0 | 1 |
|  | Sum | 100 | 55 | 67 | 54 | 49 |
