# Supplementary material for: Categorizing diffuse parenchymal lung disease in children
Source: Orphanet J Rare Dis. 2015 Sep 25;10:122. doi: 10.1186/s13023-015-0339-1 (PMC4582630; doi:10.1186/s13023-015-0339-1)
Supplement: Additional file 6: Table S5. — Centers and physicians contributing cases to the kids-lung-register. (DOCX 34 kb) [file 13023_2015_339_MOESM6_ESM.docx]

Supplemental Table 5. Centers and physicians contributing cases to the kids-lung-register

| Adler | Fribourg | Switzerland | Bouikidis | Essen |  | Erler | Dortmund |  |
| --- | --- | --- | --- | --- | --- | --- | --- | --- |
| Ahrens | Hamburg |  | Brand | Frankfurt |  | Escribano | Valencia | Spain |
| Ahrens | Lübeck |  | Brasch | Bielefeld |  | Faas | Giessen |  |
| Ahrens | Darmstadt |  | Braun | Erfurt |  | Fanconi | Zürich | Switzerland |
| Albrecht | Essen |  | Brcic | Zagreb | Croatia | Felgentreff | Freiburg |  |
| Alfaré | Uster | Switzerland | Briassoulis | Heraklion |  | Firnhaber | Hamburg |  |
| Anani | Nürnberg |  | Brodt | Frankfurt |  | Förster | Hannover |  |
| Andree | Krefeld |  | Buchenroth | Bonn |  | Freihorst | Aalen |  |
| Ankermann | Kiel |  | Buchvald | Kopenhagen | Denmark | Freisinger | Reutlingen |  |
| Armbruster | München |  | Campo | Pavia | Italy | Frommhold | Heidelberg |  |
| Aschmann | Dresden |  | Casaulta | Bern | Switzerland | Fuchs | Ulm |  |
| Baden | Tübingen |  | Chevret | Le Kremlin Bicetre | France | Gappa | Hannover |  |
| Barbato | Padova | Italy | Corbelli | Geneve | Switzerland | Gappa | Wesel |  |
| Baretton | Dresden |  | Costabel | Essen |  | Garhammer | München |  |
| Barikbin | Berlin |  | De Blic | Paris | France | Gascon | Frankfurt |  |
| Barker | Berlin |  | Delbeck | Krefeld |  | Gerein | Frankfurt |  |
| Bauer | Essen |  | Deppermann | Erfurt |  | Gerstlauer | Augsburg |  |
| Bendstrup | Aarhus | Denmark | Di Rocco | Genoa | Italy | Gesierich | Gauting |  |
| Berger | Luzern | Switzerland | Dick | Rendsburg |  | Giese | Berlin |  |
| Bernet-Büttiker | Zürich | Switzerland | Donato | Strassbourg | France | Glöckler | Erlangen |  |
| Bewig | Kiel |  | Dötsch | Köln |  | Goelz | Tübingen |  |
| Boelke | Villingen-Schwenningen | | Ebbecke | Lingen |  | Gortner | Homburg |  |
| Bohnhorst | Hannover |  | Eberle | Zürich | Switzerland | Griese | München |  |
| Borie | Paris | France | Egermann | München |  | Gröbner | Linz | Austria |
| Bosch | Karlsruhe |  | Elnazir | Dublin | Ireland | Groebel | Detmold |  |
| Bösing | Bielefeld |  | Enaud | St. Pierre | La Réunion | Grolle | Hamburg |  |
| Boske | Tuebingen |  | Engelhardt | Landshut |  | Große-Onnebrink | Essen |  |
|  |  |  |  |  |  |  |  |  |
| Grychtol | Freiburg |  | Jung | Kiel |  | Langenhorst | Salzburg | Austria |
| H.Vier | Leipzig |  | Junghänel | Köln |  | Langlitz | Iserlohn |  |
| Hammer | Basel | Switzerland | Kaiser-Labusch | Bremen |  | Lasch | Bremen |  |
| Hampel | Regensburg |  | Karen | Tuebingen |  | Latzin | Basel | Switzerland |
| Hansen | Hannover |  | Kehm | Hemer |  | Lau | Berlin |  |
| Hanssler | Essen |  | Kemen | Hamburg |  | Laux | Hamburg |  |
| Härtling | Freiburg |  | Kitz | Frankfurt am Main | | Lebecque | Brussels | Belgium |
| Hartmann | Erlangen |  | Klaer Hlawetsch | Mainz |  | Leis | Erlangen |  |
| Hecht | Frankfurt |  | Kleinert | Berlin |  | Lemke | Hamburg |  |
| Hermon | Wien | Austria | Knol | Amsterdam | Netherlands | Lenzana Fernandez | Mexico D.F. | Mexico |
| Herren | Bern | Switzerland | Köhler | Schmallenberg |  | Lex | Halle |  |
| Herrmann | Sankt Augustin |  | Kopp | Freiburg |  | Lieb | Frankfurt am Main | |
| Hinrichs- alt | Hamburg |  | Körner-Rettberg | Bochum |  | Lingenbauer | Hamburg |  |
| Hofer | Winterthur | Switzerland | Korsch | Köln |  | Littek-Rottmann | Lemgo |  |
| Höhn | Düsseldorf |  | Köster | Oldenburg |  | Litterst | Hemer |  |
| Holbe | Berlin |  | Kramer | Maastricht | Netherlands | Lohse | Singen |  |
| Holzinger | München |  | Krause | Kiel |  | Luisetti | Pavia | Italy |
| Hoppe | Köln |  | Kremers | Aalen |  | Magdorf | Berlin |  |
| Huber | Zürich | Switzerland | Kriebel | Göttingen |  | Mahlert | Oldenburg |  |
| Hülskamp | Münster |  | Kristensen | Kopenhagen | Denmark | Maier | Bern | Switzerland |
| Hünseler | Köln |  | Krüger | Freiburg |  | Mannfeld | Augsburg |  |
| Huttegger | Salzburg | Austria | Kühr | Karlsruhe |  | Manzke | Neubrandenburg |  |
| Hutten | Amsterdam | Netherlands | Kumpf | Tübingen |  | Matasova | Martin | Slovakia |
| Illing | Stuttgart |  | Kunde | Osnabrück |  | Mayr | Memmingen |  |
| Irani | Aarau | Switzerland | Kunzmann | Würzburg |  | Merz | Darmstadt |  |
| Irnstetter | München |  | Lange | Göttingen |  | Meyer | Homburg |  |
| Jong de | Amsterdam | Netherlands | Lange | Bonn |  | Miera | Berlin |  |
|  |  |  |  |  |  |  |  |  |
| Mildenberger | Mainz |  | Proesmans | Leuven | Belgium | Sanen | Leuven | Belgium |
| Mornand | Genf | Switzerland | Pross | Stuttgart |  | Sauerbrey | Erfurt |  |
| Mostafa | Wien | Austria | Ramalho | Lisboa | Portugal | Saur | Aalen |  |
| Muench | München |  | Rau | Hannover |  | Schäfer | Hamburg |  |
| Nachbaur | Wien | Austria | Reinhard | Mannheim |  | Schaible | Mannheim |  |
| Nährlich | Giessen |  | Reitz | Berlin |  | Schebeck | Kassel |  |
| Nielsen | Arhus | Denmark | Renner | Neuburg/Donau |  | Schelstraete | Gent | Belgium |
| Niesytto | Greifswald |  | Reverdin | Genf | Switzerland | Schenk | Augsburg |  |
| Nöh | Worms |  | Richter | Hannover |  | Schindera | Karlsruhe |  |
| Nüßlein | Bochum |  | Riedel | Hamburg |  | Schindler | Regensburg |  |
| O'Brien | Long Beach, CA | USA | Riedler | Schwarzach im Pongau | Austria | Schirmer-Zimmermann | Nürnberg |  |
| Ocker | Stuttgart |  | Rietschel | Köln |  | Schmitt-Grohé | Bonn |  |
| Ollerieth | Wien | Austria | Rigourd | Paris | France | Schneider | Frankfurt |  |
| Omran | Münster |  | Rochat-Guignard | Lausanne | Switzerland | Schoor van der | Amsterdam | Netherlands |
| Oppermann | Erlangen |  | Rodeck | Osnabrück |  | Schröder | Lüneburg |  |
| Ott | Münster |  | Rodriguez Becerra | Sevilla | Spain | Schroten | Mannheim |  |
| Perez Gallofre | Escalades | Andorra | Rollow | Dresden |  | Schroth | Erlangen |  |
| Peros-Golubicic | Zagreb | Croatia | Romano | Paris | France | Schuerman | Amsterdam | Netherlands |
| Petri | Neunkirchen |  | Rose | Frankfurt |  | Schulze | Frankfurt |  |
| Pfleger | Graz | Austria | Rosenthal | London | England | Schuster | Düsseldorf |  |
| Pilgrim | Luzern | Switzerland | Roth | Köln |  | Schwerk | Hannover |  |
| Pin | Grenoble | France | Rücker | München |  | Seidenberg | Oldenburg |  |
| Pinheiro | Barga | Portugal | Ruf | Mannheim |  | Silwedel | Würzburg |  |
| Poplawska | Mainz |  | Runge | Wuppertal |  | Singer | Hamburg |  |
| Pöschl | Heidelberg |  | Rupprecht | Zollikerberg |  | Slot | Amsterdam | Netherlands |
| Prenzel | Leipzig |  | Ruß | Köln |  | Sommerburg | Heidelberg |  |
| Probst | Freiburg |  | Saadi | München |  | Stanzel | Gauting |  |
| Stehling | Essen |  | Wiebe | Sankt Augustin |  | Steiß | Giessen |  |
| Steinhagen | Wuppertal |  | Wiebel | Heidelberg |  | Stengel | Aalen |  |
| Stocker | Luzern | Switzerland | Wildhaber | Zürich | Switzerland |  |  |  |
| Stöhring | Berlin |  | Willasch | Frankfurt |  |  |  |  |
| Störmann | Osnabrück |  | Wintgens | Moenchengladbach | |  |  |  |
| Stumpner | Eschwege |  | Wittekindt | Frankfurt |  |  |  |  |
| Teig | Bochum |  | Woitsch | München |  |  |  |  |
| Teschler | Essen |  | Wolff | Mannheim |  |  |  |  |
| Thomas | Würzburg |  | Yalcin | Ankara | Turkey |  |  |  |
| Ueköter | Münster |  | Zeidler | Bonn |  |  |  |  |
| van de Loo | Amsterdam | Netherlands | Zielen | Frankfurt |  |  |  |  |
| van Dellen | Amsterdam | Netherlands | Zimmer | Gießen |  |  |  |  |
| van Kaam | Amsterdam | Netherlands | Zimmermann | Erlangen |  |  |  |  |
| Varnholt | Berlin |  |  |  |  |  |  |  |
| Vierzig | Köln |  |  |  |  |  |  |  |
| Vogelberg | Dresden |  |  |  |  |  |  |  |
| Vollmann | Neuwied |  |  |  |  |  |  |  |
| von der Hardt | Hannover |  |  |  |  |  |  |  |
| von der Thüsen | Amsterdam | Netherlands |  |  |  |  |  |  |
| Weckelmann | Wuppertal |  |  |  |  |  |  |  |
| Weis | Koblenz |  |  |  |  |  |  |  |
| Weitzdoerfer | Wien |  |  |  |  |  |  |  |
| Wellmann | Basel | Switzerland |  |  |  |  |  |  |
| Welzing | Köln |  |  |  |  |  |  |  |
| Werner | Münster |  |  |  |  |  |  |  |
| Welzing | Köln |  |  |  |  |  |  |  |
| Werner | Münster |  |  |  |  |  |  |  |
